# The effect of one-step and multi-step polishing systems on surface texture of two different resin composites

**DOI:** 10.4103/0972-0707.62637

**Published:** 2010

**Authors:** Kusum Bashetty, Sonal Joshi

**Affiliations:** Department of Conservative Dentistry and Endodontics, The Oxford Dental College and Hospital Bommanahalli, Hosur Road, Bangalore - 560 068, India; 1 Department of K.L.E.S's Institute of Dental Sciences and Research Centre, Belgaum - 590010, India

**Keywords:** Finishing and polishing systems, PoGo one-step polishing systems, super snap polishing system, profilometer

## Abstract

**Objective:**

The purpose of this *in-vitro* study was to evaluate the surface roughness of two direct resin composites polished with one-step and multi-step polishing systems.

**Materials and Methods:**

The resin composites examined in this study include minifill-hybrid composite Esthet-X (DENTSPLY/Caulk, Milford, DE, USA) and packable composite Solitaire II (Heraeus Kulzer, Inc., Southbend). A total of 42 discs (10 × 2 mm), 21 specimens of each restorative material were fabricated. Seven specimens per composite group received no polishing treatment and served as control. For each composite group, the specimens were randomly divided into two polishing systems: One-step PoGo (Dentsply/Caulk, Milford, DE, USA) and multi-step Super Snap (Shofu, Inc. Kyoto, Japan). Polishing systems were applied according to the manufacturer's instructions after being ground wet with 1200 grit silicon carbide paper. The surface roughness values were determined using a profilometer.

**Results:**

Data was subjected to student's *t* test at a significance level of 0.05. The smoothest surfaces were achieved under Mylar strips in both the composite groups. Mean Ra values ranged from 0.09 to 0.3 μm for Esthet-X group and from 0.18 to 0.3 μm for Solitaire II with different polishing systems. The ranking of the order of surface roughness on the basis of the type of composite was as follows: Esthet-X < Solitaire II for PoGo system and Esthet-X = Solitaire II for Super Snap; and the ranking for the polishing system was: PoGo < Super Snap (*P* ≤ 0.05).

**Conclusion:**

The one-step polishing system (PoGo) produced better surface quality in terms of roughness than the multi-step system (Super Snap) for minifill-hybrid composite (Esthet-X), and it was equivalent to Super Snap for packable composites (Solitaire II). Minifill-hybrid presented a better surface finish than Solitaire II when PoGo polishing system was used. No significant difference was present in surface roughness between both the materials when Super Snap system was used.

## INTRODUCTION

Composite resins are one of the most commonly used direct restorative materials. Its clinical use has expanded considerably over the past few years because of the increased esthetic demand by patients, new developments in formulations and simplification of bonding procedures.[1] Regardless of the cavity class and location, a smooth surface finish is clinically important as it determines the esthetics and longevity of composite restoration. The presence of surface irregularities arising from poor finishing/polishing techniques and/or instruments can create staining, plaque retention,[2] gingival irritation, recurrent caries, abrasivity, wear kinetics[3–4] and tactile perception.[5] Furthermore, a smooth surface adds to the patient's comfort as a change in surface roughness of 0.3 μm can be detected by the tip of the tongue.[5]

Composites polymerized with a clear matrix on the surface will leave a resin-rich surface layer that is easily abraded in the oral environment, exposing unpolished, rough, inorganic filler material. Since such a finish cannot be maintained, further contouring and finishing is required.

Finishing is defined as the gross contouring or reduction of a restoration to obtain ideal anatomy. Polishing refers to the reduction in roughness and scratches created by finishing instruments.[6] A variety of instruments such as carbide and diamond burs, abrasive finishing strips and polishing pastes may be frequently used to finish tooth-colored restorative materials.[7–10] Clinicians have their choice among a wide range of finishing and polishing instruments. Previously, significant importance was given the application of progressively finer grits of abrasives to polish resin composite restorations. Today, many attempts have been made to develop composite finishing instruments that are suitable for all four steps of the trimming procedure.[11] Current one-step systems appear to be as effective as multi-step systems for polishing dental composites.[1] With the ultimate goal of achieving a smooth surface of the composite restoration in fewer steps, the one-step polishing systems are appealing to the clinician. Because of the variety of composites and polishing systems available, they should be evaluated in order to verify which polishing systems yields the best polishing effect on a given composite. The purpose of this study was two fold: (1) To study the polishing effect of two different polishing systems: One-step (PoGo) and multi-step (Super Snap) on two different composites; and (2) To determine the surface finish of minifill-hybrid (Esthet-X) and packable (Solitaire II) composites and whether filler size and content have any effect on it.

## MATERIALS AND METHODS

The resin composites examined in this study were Esthet-X (DENTSPLY/Caulk, Milford, DE, USA) and Solitaire II (Heraeus Kulzer, Inc Southbend). Table 1 shows the composition and manufacturers of the materials.

**Table 1:** Composition and manufacturers of the tested resin composites

Resin composites	Composition	Manufacturers
Esthet-X (minifill-hybrid)	Bariumalumino fluoroborosilicate glass (BAFG) and nano-sized silicon dioxide particles (0.85-0.9 μm, 77% wt) Modified Bis-GMA, TEGDMA, UDMA	Dentsply/Caulk, Milford, DE, USA
Solitaire II (packable composite)	Silicon dioxide, BAFG, aluminum fluoride glass Indirect high heat and pressure cured polycarbonate vitroid glass ceramic material (2-20 μm, 80%-85% wt)	Heraeus Kulzer, Inc. Southbend, USA

The finishing/polishing systems tested were PoGo (DENTSPLY/Caulk, Milford, DE, USA) and Super Snap (Shofu Inc., Kyoto, Japan). Table 2 shows the composition, manufacturers and sequence of the tested polishing system.

**Table 2:** Composition, manufacturers and sequences of the investigated polishing systems

Polishing systems	Composition	Manufacturers	Usage	Handpiece speed
Super snap (multi-step)	Aluminum oxide	Shofu, inc, Kyoto, Japan	Dry, 6 strokes coarse	12,000 rpm
			Dry, 6 strokes medium	12,000 rpm
			Dry, 6 strokes fine	12,000 rpm
			Dry, 6 strokes extra-fine	12,000 rpm
			Dry, 6 strokes coarse	
PoGo (one-step)	Polymerized urethane dimethacrylate resin, Fine diamond powder, silicon oxide	Dentsply/Caulk, Milford, DE, USA	Dry, 24 light intermittent strokes	12,000 rpm

A total of 42 specimens (21 specimens of each restorative material) were fabricated using a customized cylindrical rubber base mould (10 × 2 mm). The resin composites were placed using a plastic instrument and covered with a mylar strip. A 1-2 mm thick glass slide was placed over the strip before curing with the light activating source (tungsten-halogen lamp, Trilight, 3M, ESPE, Seefeld, Germany) to flatten the surfaces. The samples were then cured for 40s through the mylar strips and the glass slide. The curing light intensity was measured at 800mW/cm^2^ and monitored using a built-in radiometer. An additional 20 s curing was done on both the sides of the specimens after removing the strips and glasses. The cured samples were then stored in 100% humidity at 37°C for 24 h prior to the finishing procedures.

To reduce variability, all specimen preparation and finishing/polishing procedures were performed by the same operator. The specimens were randomly separated into three treatment groups (n = 7). The Mylar strip groups were selected, and others were wet ground with 1200 grit silicon carbide paper to provide a base line before using the polishing systems. A slow-speed handpiece rotating at a maximum 12,000 rpm was used with a constant moving repetitive stroking action to prevent heat build up and the formation of grooves. A new polishing disc was used for each specimen and was discarded after each use.

The groups were divided as follows for each composite:

Group I: (Control) – Mylar strip

Group II: Super snap discs

Group III: PoGo discs

Table 2 reflects the details of the finishing and polishing sequence that were based on the manufacturer's instructions. The polished resin composite discs were washed, allowed to dry and kept again in 100% humidity for 24 h before measuring the average surface roughness values (Ra). The surface roughness test was performed using a profilometer (Surftest-211, Mitutoyo; Kanagava, Japan). Readings were taken at the centre of each specimen, and four sampling lengths of 0.25 mm each were used, giving a total evaluation length of 1 mm. The data were analysed using student's *t* test at a significance level of 0.05.

## RESULTS

Table 3 shows the mean surface roughness observed with different finishing and polishing systems. Results of statistical analysis are shown in Table 4. Mean Ra values ranged from 0.09 to 0.3 μm for Esthet-X and from 0.18 to 0.3 μm for Solitaire II with different polishing systems. For both the materials, the smoothest surface was obtained with mylar strip and the roughest with Super Snap. The lowest Ra values obtained for Esthet-X and Solitaire II were 0.09 μm and 0.18 μm, respectively.

**Table 3:** Mean surface roughness with different finishing/polishing systems

Finishing/polishing system	Esthet-X Ra ± SD	Solitaire II Ra ± SD
Mylar strip (control)	0.09 ± 0.04	0.18 ± 0.08
Super snap	0.3 ± 0.1	0.3 ± 0.07
PoGo	0.19 ± 0.06	0.25 ± 0.07

SD = Standard deviation

**Table 4:** Comparison of mean surface roughness between finishing/polishing systems

Materials	Differences
Esthet-X	Mylar strip < PoGo < Super snap
Solitaire II	Mylar strip < PoGo, Super snap

< denotes statistically significant difference (*P* ≤ 0.05)

For Esthet-X, specimens treated with PoGo were found to be significantly smoother than specimens treated with Super Snap. In Solitaire II group, no significant difference in Ra values was observed between PoGo and Super Snap.

On comparing Solitaire II and Esthet-X, the latter provided a significantly smoother surface when PoGo polishing system was used. Super Snap polishing system produced the same surface roughness for both the materials.

## DISCUSSION

A resin composite restoration can be imperceptible to the naked eyes when its surface closely resembles the surrounding enamel surface. Thus, polished restoration should demonstrate an enamel-like surface texture and gloss. In this study, the smoothest surfaces were obtained by curing both materials against a matrix strip. This finding was in agreement with previous studies[6,12–14] on resin composites. Although the surface obtained with a mylar strip is perfectly smooth, it is rich in resin organic binder. Therefore, the removal of the outermost resin by finishing and polishing procedure would tend to produce a harder,[15] more wear resistant and hence a more esthetically stable surface.[14] Therefore, it is clinically important to determine the finishing techniques that result in the smoothest surface with minimum time and instruments.

Improper application of finishing/polishing instruments could lead to decreased effectiveness and less than optimal results.[16] Strict adherence to manufacturers' instructions on finishing/polishing procedures was thus observed. Efforts were also made to standardize the different aspects of the methodology including handpiece speed, type of motion used and the total number of strokes employed for each finishing/polishing system. The slight variations in Ra values with-in each treatment group may be accounted for by the unequal distribution of abrasives in the delivery medium and the differences in pressure exerted during finishing/polishing procedures. The differences in pressure exerted during finishing/polishing procedures were minimized by using a single operator for the experiment. The term finishing/polishing was employed instead of finishing and polishing, as the two processes are inter-related and cannot be easily demarcated.

According to Stoddard and Johnson,[14] the effectiveness of finishing/polishing systems depends on material (filler size and content), type of abrasive used, time spent with each abrasives, strokes, amount of pressure applied, orientation of abrading surfaces and geometry (discs, cups, cones) of abrasive instruments.

For years, specially designed diamonds with very fine abrasive particle size and white Arkansas stones have been used to polish resin composite restorations.[17] However, the use of diamond burs is limited to initial contouring because of their ability to remove equal amount of adjacent enamel.[18] Later, importance was given to the application of progressive finer grits of abrasives to polish resin composites with little concern for the type of motion employed during their use.

Fruits and others,[11] have reported that three types of motion may be equally critical to the development of optimal surface smoothness: A rotary motion (circular), a planar motion and a reciprocating motion. They concluded that, for all possible combinations of materials and abrasive grits, the planar motion achieved the lowest roughness values. In the current study, a planar motion was used for both the polishing systems.

Most investigators have concluded that flexible aluminum oxide disks are the best instruments for providing low roughness on composite surfaces.[6,19,20] However, aluminum oxide disks (Super snap) have limitations due to their geometry. While using the disks, it is often difficult to efficiently create, finish and anatomically polish contoured surfaces, especially in the posterior region of the mouth.

To reduce the clinical time, the technology for two and one-step finishing/polishing system has evolved over the past few years and current systems appear to be as effective as multi-step systems for finishing and polishing dental composites. The obvious advantage of the one-step system is the convenience and efficiency of producing a very smooth surface without having to switch to finer polishing items or having to wash and dry between each step to ensure removal of the larger abrasives from the previous step.

For this study, PoGo is used as a one-step micro-polisher, but the manufacturer recommends pre-treatment with the Enhance system to obtain favorable results. As per the definition, PoGo is a one-step method and indeed, some authors use this system without any pre-treatment.[1,21] For this reason, the authors of this study classified and applied PoGo as a one-step method.

The surface micro-morphology of resin composite after finishing and polishing has been shown to be influenced by the size, hardness and amount of filler particles.[22] In this study, the surface finish of minifill-hybrid composite (Esthet-X) was significantly better than packable composite (Solitaire II) when PoGo system was used. In composites, where the filler particles are significantly harder than the matrix, the resin phase may suffer a preferential loss during finishing and polishing. This will result in the filler phase showing positive relief on the surfaces.[6,22] Materials with larger and harder filler particles are therefore expected to have higher Ra values after finishing/polishing. The filler particle size of Esthet-X ranged from 0.85 to 0.9 μm, while that of Solitaire II ranged from 2 to 20 μm. In view of the aforementioned, the significantly higher Ra values observed with Solitaire II after finishing/polishing is expected.[6]

According to Marigo and others,[23] the final glossy surface obtained by polishing depends on the flexibility of the backing material in which the abrasive is embedded, the hardness of the particles, the instruments and their geometry (cups, discs and cones). In this study, PoGo achieved a smoother surface compared to Super Snap for Esthet-X group. This result is in accordance with that of previous studies.[21] The superior performance of PoGo may be attributed in part to the use of fine diamond powders instead of aluminum oxide (Super Snap) and the cured urethane dimethacrylate resin delivery medium. According to Roeder,[15] aluminum oxide disks produced the smoothest surface for packable composites (Solitaire II). In this study, no significant difference was observed between PoGo and Super Snap for Solitaire II group. Results of this study correlate well with that of previous studies.[21,24] The PoGo system is very sensitive to the mode of application because it requires polishing at two different loads. Therefore, by using this system, inter-individual differences with respect to manual application and polishing could have a greater effect on results than is the case of the other method.

Many studies on the polishing of resin composites have been introduced and the most commonly used parameter to describe surface roughness is Ra.[3,15,20] Surface roughness is a function of the microstructure created by the series of physical processes used to modify the surface and is related to the scale of the measurement. Profilometers have been used for years to measure the surface roughness for *in-vitro* investigations. They provide limited two-dimensional information. However, an arithmetic average roughness can be calculated and used to represent various material-polishing surface combinations that assist clinicians in their treatment decisions.[23,25] However, the complex structure of a surface cannot be fully characterized by the use of only surface roughness measurements. Therefore, it is not appropriate to draw conclusions on the clinical suitability of a finishing instrument exclusively on the basis of roughness average results. However, in combination with atomic force microscopy (AFM)[26] and scanning electron microscope (SEM) analysis that permits evaluation of the destructive potential of the finishing tool, more valid predictions of clinical performance can be made.[26] In this study, surface roughness measurements were used only for relative comparisons.

Further studies are needed to determine which finishing technique is best suited to clinical situations, where access is limited and restoration surfaces are not flat along with AFM and SEM analysis to obtain more valid results.

## CONCLUSION

Within the limitations of the study, the following conclusions are obtained:

- Mylar strip produced the smoothest of all the finishing and polishing systems. All finishing/polishing procedures decreased the smoothness obtained with matrix strips and resulted in Ra values above the threshold value of 0.3 μm.
- The effect of finishing/polishing systems on surface roughness was material dependent.
- For Esthet-X composite, PoGo provided significantly smoother surface than Super Snap.
- For Solitaire II composite, no significant differences in surface roughness were observed between PoGo and Super Snap.
- Surface finish of minifill-hybrid composite (Esthet-X) was significantly better than packable composite (Solitaire II) when PoGo polishing system was used.
- No significant difference was present in surface roughness between both the materials when Super Snap system was used.
- Considering the reduced steps, application time and the elimination of cross-infection risks, one-step polishing systems may be preferred for polishing resin composites.

## References

[1] Yap AU, Yap SH, Teo CK, Ng JJ (2004). Finishing/polishing of composite and Compomer restoratives: Effectiveness of one-step systems. Oper Dent.

[2] Kawai K, Urano M (2001). Adherence of plaque components to different restorative materials. Oper Dent.

[3] Mandikos MN, McGivney GP, Davis E, Bush PJ, Carter JM (2001). A comparison of the wear resistance and hardness of indirect composite resins. J Prosthet Dent.

[4] Tjan AH, Chan CA (1989). The polishibility of posterior composites. J Prosthet Dent.

[5] Jones CS, Billington RW, Pearson GJ (2004). The *in-vivo* perception of roughness of restoration. Br Dent J.

[6] Yap AU, Lye KW, Sau CW (1997). Surface characteristic of tooth coloured restoration polished utilizing different polishing systems. Oper Dent.

[7] Jefferies SR (1998). The art and science of abrasive finishing and polishing in restorative dentistry. Dent Clin North Am.

[8] Attar N (2007). The effect of finishing and polishing procedures on the surface roughness of composite resin materials. J Contemp Dent Pract.

[9] Uçtaşli MB, Bala O, Güllü A (2004). Surface roughness of flowable and packable composite resin materials after finishing with abrasive discs. J Oral Rehabil.

[10] Türkün LS (2004). Effect of re-use of a disposable micro-polisher on the surface of a microhybrid resin composite. Am J Dent.

[11] Fruits TJ, Miranda FJ, Coury TL (1996). Effects of equivalent abrasive grit sizes utilizing differing polishing motions on selected restorative materials. Quintessence Int.

[12] Wilson F, Heath JR, Watts DC (1990). Finishing composite restorative material. J Oral Rehabil.

[13] Tate WH, Powers JM (1996). Surface roughness of composites and hybrid ionomers. Oper Dent.

[14] Stoddard JW, Johnson GH (1991). An evaluation of polishing agents for composite resins. J Prosthet Dent.

[15] Roeder LB, Tate WH, Powers JM (2000). Effect of finishing and polishing procedures on the surface roughness of packable composites. Oper Dent.

[16] Kanter J, Koski RE, Bogdan MS (1983). How to achieve the best surface polish on composite resin. CDA J.

[17] Roulet JF, Hirt T, Lutz F (1984). Surface roughness and marginal behaviour of experimental and commercial composites: An *in vitro* study. J Oral Rehabil.

[18] Quiroz L, Lentz DL (1986). The effect of polishing procedures on the surface smoothness of several light-cured posterior composites. Compend Contin Educ Dent.

[19] Setcos JC, Tarim B, Suzuki S (1999). Surface finish produced on resin composites by new polishing systems. Quintessence Int.

[20] Hoelscher DC, Neme AM, Pink FE, Hughes PJ (1998). The effect of three finishing systems on four aesthetic restorative materials. Oper Dent.

[21] St-Georges AJ, Bolla M, Fortin D, Muller-Bolla M, Thompson JY, Stamatiades PJ (2005). Surface finish produced on three resin composites by new polishing systems. Oper Dent.

[22] Chung KH (1994). Effect of finishing and polishing procedure on the surface texture of resin composites. Dent Mater.

[23] Marigo L, Rizzi M, La Torre G, Rumi G (2001). 3-D surface profile analysis: Different finishing methods for resin composites. Oper Dent.

[24] Ryba TM, Dunn WJ, Murchison DF (2002). Surface roughness of various packable composites. Oper Dent.

[25] McLundie AC, Murray FD (1974). Comparison of methods used in finishing composite resin - A SEM study. J Prosthet Dent.

[26] Kakaboura A, Fragouli M, Rahiotis C, Silikas N (2007). Evaluation of surface characterstics of dental composites using Profilometery, Scanning electron microscopy and Gloss-meter. J Mater Sci Mater Med.

